# Imidacloprid exposure cause the histopathological changes, activation of TNF-α, iNOS, 8-OHdG biomarkers, and alteration of caspase 3, iNOS, CYP1A, MT1 gene expression levels in common carp (*Cyprinus carpio* L.)

**DOI:** 10.1016/j.toxrep.2017.12.019

**Published:** 2017-12-27

**Authors:** Selçuk Özdemir, Serdar Altun, Harun Arslan

**Affiliations:** a Department of Genetics, Faculty of Veterinary Medicine, Atatürk University, Yakutiye, 25240, Erzurum, Turkey; b Department of Pathology, Faculty of Veterinary Medicine, Atatürk University, Yakutiye, 25240, Erzurum, Turkey; c Department of Basic Sciences, Faculty of Fisheries, Atatürk University, Yakutiye, 25240, Erzurum, Turkey

**Keywords:** IMI, Imidacloprid, iNOS, inducible Nitric Oxide Synthase, 8-OHdG, 8-hydroxy-2-deoxyguanosine, TNF-α, Tumor Necrosis Factor-α, CYP1A, Cytochrome P450 1A, MT1, Metallothionein 1, IF, Immunofluorescence, H&E, Hematoxylin and Eosin, Imidacloprid, Common carp, iNOS, 8-OHdG, TNF-α, Caspase 3

## Abstract

Imidacloprid (IMI) is a neonicotinoid that is widely used for the protection of crops and carnivores from insects and parasites, respectively. It is well known that imidacloprid exposure has a harmful effect on several organisms. However, there is little information about imidacloprid toxicity in aquatic animals, particularly fish. Thus, in the current study, we assessed the histopathological changes; activation of iNOS, 8-OHdG and TNF-α; and expression levels of caspase 3, iNOS, CYP1A and MT1 genes in the common carp exposed to imidacloprid. For this purpose, fish were exposed to either a low dose (140 mg/L) or a high dose (280 mg/L) of imidacloprid for 24 h, 48 h, 72 h and 96 h. After IMI exposure, we detected hyperplasia of secondary lamellar cells and mucous cell hyperplasia in the gills, as well as hydropic degeneration in hepatocytes and necrosis in the liver. Moreover, 8-OHdG, iNOS and TNF-α activation was found particularly in the gills and liver but also moderately in the brain. Transcriptional analysis showed that caspase 3 expression was altered low dose and high doses of IMI for 72 h and 96 h exposure (*p <* 0.05), iNOS expression was up-regulated with both low and high doses of IMI and in a time-dependent manner (*p < *0.05, *p <* 0.01, *p < *0.001), CYP1A expression was not significantly changed regardless of the dose of IMI and exposure time (*p > *0.05) except with low and high doses of IMI for 96 h (*p <* 0.05), and lastly, MT1 gene expression was up-regulated only in the brain with low doses of IMI for 96 h and high doses of IMI for 48 h, 72 h and 96 h exposure (*p <* 0.05, *p <* 0.01). Our results indicated that acute IMI exposure moderately induce apoptosis in the brain but caused severe histopathological lesions, inflammation, and oxidative stress in the gills, liver, and brain of the common carp.

## Highlights

• IMI toxication was evaluated with three different methods.
• Pathological lesions were observed after IMI exposure in gills, liver and brain.
• IMI exposure induced iNOS, 8-OHdG and TNF-α activation in gills, liver and brain.
• IMI exposure caused upregulation iNOS, caspase 3 and MT1 expressions in brain.

## Graphical abstract

**Figure:**
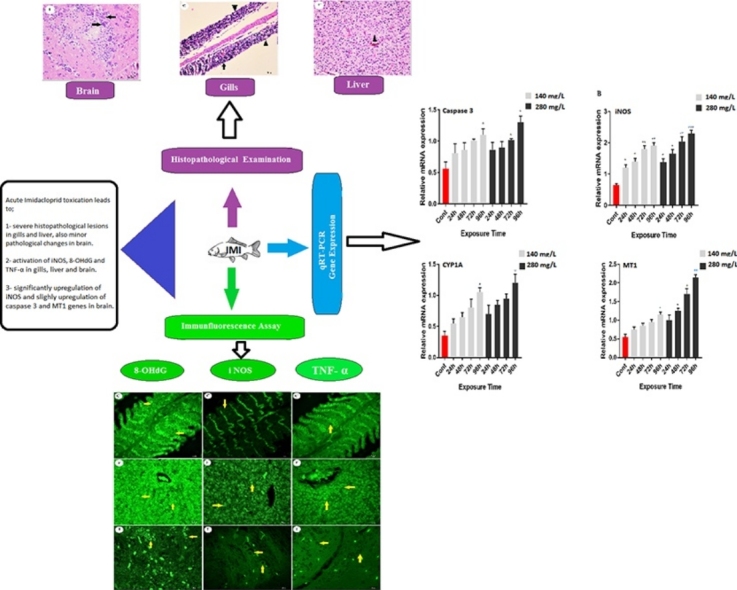

## 1 Introduction

Imidacloprid (IMI) is used as an insecticide and was first commercially produced by Bayer in 1991 [1]. IMI is also an agonist of postsynaptic nicotinic acetylcholine receptors (nAChRs), referred to as neonicotinoids, and thus, it causes impairment of the central nervous system (CNS) [2]. nAChRs regulate cholinergic synaptic transmission [3]. IMI is used to destroy insects infesting crops and parasites of carnivores [4]. The use of imidacloprid has been a controversial issue since the European Food Safety Authority reported its highly toxic effects on bees and farmers. There are different reports on the environmental toxicity of IMI. The compound (IMI) was selected based on high use, prior linkage with aquatic toxicity in the region, or emerging use with little previous monitoring. Although some studies claimed that IMI does not leach into groundwater, some other studies suggested the opposite [[5], [6], [7]]. IMI can spread with storms, run-off and spray drift after its application. Therefore, the potential adverse effects of IMI exposure in non-target organisms, including humans, animals, and particularly aquatic animals, is of increased interest. Recently, a few studies have investigated IMI toxicity in aquatic organisms due to its increased use; moreover, there are few data on apoptosis and immunotoxicity mechanisms concerning IMI toxicity in aquatic organisms [8]. Hence, it is necessary to assess IMI toxicity in vertebrate fish. This is also important for non-target organisms such as humans and animals.

IMI toxicity leads to harmful mutagenic and carcinogenic effects in humans and animals [9]. Long-term IMI exposure induce the genotoxic effects and oxidative stress in rabbits [[10], [11], [12]]. In the previous study, researchers was applied for the monitoring of IMI and 6-chloronicotinic acid (6-ClNA) with LC-APCI-MS based method in hair and urine of rabbits intentionally fed with insecticide at low or high doses for 24 weeks [13]. IMI could be cause the hepatotoxicity and nephrotoxicity at a dose much lower than the LD50 in mice [14]. Moreover, the mixture of neonicotinoid, organophosphate and herbicide cause the oxidative injury in zebrafish [15]. Inflammation, oxidative stress, DNA damage and apoptosis are induced by several environmental factors, one of which is pesticide intoxication. During pesticide intoxication, activation of some biomarkers (iNOS, 8-OHdG, TNF-α) and expression of some apoptotic genes (caspase 3) are increased [16,17]. Reactive nitrogen species (RNS) such as nitric oxide (NO), which is produced by inducible nitric oxide synthase (iNOS), endothelial nitric oxide synthase (eNOS) and neuronal nitric oxide synthase (nNOS), are also increased as a consequence of oxidative stress [18]. Pesticide toxicity induces the generation of iNOS [19]. Therefore, iNOS is used as a biomarker of inflammation and oxidative stress in toxicity studies in different organisms [20]. In addition, 8-hydroxy-2′-deoxyguanosine (8-OHdG), which is produced as a result of oxidative attack to DNA or RNA, is widely used as a biomarker of DNA damage and oxidative stress [21]. Activation of 8-OHdG has been shown to be increased in fish brains exposed to toxic chemicals [22,23]. Moreover, tumor necrosis factor-alpha (TNF-α) is an inflammatory mediator [24]. Exposure to certain insecticides can increase TNF-α activation in the blood and brain [25]. A few studies have reported that IMI toxicity induces 8-OHdG and TNF-α activation in animals [16,26]. It is not clear whether 8-OHdG and TNF-α are activated in aquatic animals, particularly fish, because the effects of IMI toxicity in these animals are poorly understood. Therefore, the investigation of how IMI toxicity affects 8-OHdG and TNF-α expression in fish will be useful for the assessment of DNA damage and aquatic animal health.

Several environmental pollutants such as pesticides can cause programmed cell death (PCD) or apoptosis in animals and humans [27,28]. Apoptosis is a crucial mechanism for the elimination of abnormal cells that can transform into cancer cells [29]. Caspase 3, which belongs to the family of cysteine-dependent aspartate proteases, is an effector protein in intrinsic and extrinsic apoptosis pathways [30]. In addition, it has been used as an apoptotic marker in several studies, including embryogenesis, cancer, developmental, and neurotoxicological research [31]. It has also been used in pesticide toxicity studies in aquatic animals [31]. Previous studies have reported that chlorpyrifos (CPF), methyl parathion (MPT), trichlorfon, and malathion (MLT) increase caspase 3 mRNA levels in rats [27]. IMI’s mechanism of toxicity involves the up-regulation of caspase 3 gene expression in rats and honey bees [32,33]. However, it is not known whether IMI also affects caspase 3 expression in the common carp. Elucidation of this issue could provide useful information on the relationship between apoptosis and IMI toxicity in the common carp. Thus, it is necessary to evaluate the harmful effect of IMI on aquatic animals’ health.

Metallothioneins (MTs) can bind to zinc and copper. MTs have been shown to eliminate the adverse effects of exposure to toxic elements, including heavy metals and pesticides, and to protect organisms from several stress conditions such as oxidative stress [[34], [35], [36]]. Pesticides can induce MT synthesis in fish [37]. Cytochrome P450 isoforms play a role in the metabolism of xenobiotics such as pesticides [38,39], while they are also responsible for the metabolism of pollutants, drugs and several carcinogenic chemicals [40]. There are several types of Cytochrome P450 isoforms, one of which is CYP1A [41]. MTs and CYP1A syntheses are related to reactive oxygen species (ROS), and thus, they can be used as oxidative stress biomarkers [42]. Little information is available on the expression of MT1 and CYP1A in response to pesticide intoxication in aquatic animals. However, no data are available on whether IMI exposure changes the expression of MT1 and CYP1A genes in the brain of the common carp. For this reason, it is necessary to investigate the mechanism of IMI toxicity in the common carp.

Since there are no available studies on IMI toxicity in the common carp, we investigated the potential toxic effects of IMI and assessed inflammation, oxidative stress, DNA damage and apoptosis associated with IMI in this animal model. For this purpose, we first evaluated the histopathological changes due to IMI toxicity by histopathological examination of gills, liver and brain. Subsequently, the activation of iNOS, 8-OHdG and TNF-α was assessed by immunofluorescence (IF) in the brain. Lastly, we used RT-qPCR to measure mRNA levels of caspase 3, iNOS, CYP1A and MT1 genes in the brain.

## 2 Materials and methods

### 2.1 Chemical

**Fig. 1:**
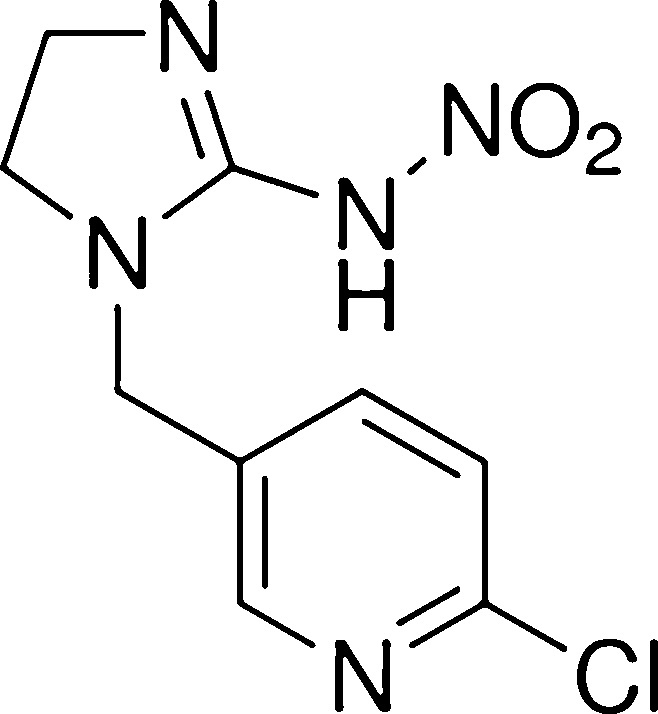
The molecular structure of Imidacloprid (IMI).

Imidacloprid (IMI), C_9_H_10_CIN_5_O_2_ (CAS Number 138261-41-3 5, ≥98% purity, molecular weight of 255.66), was purchased from Sigma-Aldrich (Germany). The molecular structure of imidacloprid is shown in Fig. 1. Stock solutions were prepared in accordance with the International Union of Pure and Applied Chemistry [43].

### 2.2 Animals and experimental design

Common carps were obtained from Atatürk University, Faculty of Fisheries and the Inland Water Fish Breeding and Research Center. This study was performed in accordance with the approved ethical rules of Atatürk University. Fish were fed for 45 days in a stock pond to achieve acclimatization to their environmental conditions. After the adaptation period, twelve fish were put in each water aquarium (volume 70 l). The fish were exposed to the IMI LD50 for periods of 24 h, 48 h, 72 h and 96 h. The tested fish had an average weight of 88 ± 1.8 g and length of 12.5 ± 0.4 cm. The physico-chemical properties of the aquarium water were the following: O2 = 8.3 ± 0.1 ppm, pH = 7.6 ± 0.2, SO4-2 = 10.32 ppm, CO3–2 = 124 ppm, HCO3- = 145.6 ppm, NO3- = 3.45 ppm, NO2- = trace, conductivity = 276 ms/cm, total hardness = 119 ppm of CaCO3, and temperature (24–330C) and water was changed every day. The fish were divided into six groups. Two aquaria were prepared for each group. Each aquarium contained 12 fish (n = 72). The fish were exposed to the chemical for periods of 96 h in static aquarium systems. The fish in groups I and II were the control. The fish in groups III and IV were administered doses of 280 mg/L (1/5 LD50), whereas those in groups V and VI were given a dose of 140 mg/L (1/10 LD50) of imidacloprid (Table 1). These doses were adjusted according to a previous study on IMI toxicity in cyprinids [44]. After 96 h, the fish were sacrificed by cervical dislocation and placed in an ice bath. Their gills, livers and brains were collected for histopathological examination, immunofluorescence assay, also brain tissues were quickly removed and stored at −80 °C for the total RNA isolation.

**Table 1:** Imidacloprid Treatment Design, 96 h.

Group	I	II	III	IV	V	VI
Treat.	Control	Control	IMI H. Dose	IMI H. Dose	IMI L. Dose	IMI L. Dose
Dose	X	X	280 mg/L	280 mg/L	140 mg/L	140 mg/L
Lethal Dose	X	X	1/5 LD_50_	1/5 LD_50_	1/10 LD_50_	1/10 LD_50_
n	12	12	12	12	12	12

### 2.3 Histopathological examination

Tissue samples were stored for 1 day to be fixed in a 10% buffered formalin solution. Routine histopathological alcohol and xylene series for samples was performed in Shandon Citadel 2000 (USA) tissue system. After the routine histopathology process, all samples were poured into paraffin for blocking and prepared microtome sections in 5 μm by using rotary microtome (Leicia RM 2255). All sections were stained with routine Haematoxylin eosin staining for histopathological evaluation. Slides were examined under the light microscopy (Olympus BX51 with DP72 camera attachment).

### 2.4 Immunofluorescence staining

After deparaffinization, 3% H_2_O_2_ solution was dropped on slides for ten minutes to inactivate endogenous peroxidase activity. Then the slides were immersed in antigen retrieval solution (ab 96674, Abcam, USA) (pH 6.0) and heated in microwave for 15 min to unmasked antigens. After waiting for cooling, sections were incubated with protein block solution (ab 80436, Abcam, USA) to prevent nonspecific bindings for 15 min. A solution (ab 64211; Abcam, USA) was used for antibody dilution. Sections were incubated with diluated antibodies (iNOS, ab15323, dilution: 1/200; Abcam, USA. 8-OHdG, sc-66036, dilution: 1/200, Santa Cruz, EU. TNFα, NBP1-19532, dilution: 1/250; Novus, USA) at room temperature for 30 min. The seconder polyclonal fluorescence antibody goat pAB to Rb IgG (FITCH) (ab 6717, dilution: 1/100, Abcam, USA) was used for sections incubated with iNOS and TNFα. The seconder monoclonal fluorescence antibody pAB to Ms IgG (FITCH) (ab 6785, dilution: 1/400, Abcam, USA) was used for sections incubated with 8-OHdG. Finally all sections were covered with lamellas by using glycerin diluated 1/9 with distilled water for evaluation under fluorescence microscopy system (Zeiss Scope A1 with Axio cam ICc5 camera attachment system).

### 2.5 Total RNA isolation and reverse transcription quantitative real-time PCR (RT-qPCR)

For the assessment of mRNA expression of caspase 3, iNOS, CYP1A and MT1, real-time PCR was used with a CFX96 TouchTM Real-Time PCR Detection System (Bio-Rad, USA). Total RNA was isolated from brain tissue using TRIZOL reagent (Invitrogen, Cat: 15596026, USA) according to the manufacturer’s instructions. The RNA samples were treated with RNase-free DNase (Thermo Scientific, Lithuania), and then cDNA synthesis was performed using a QuantiTect Reverse Transcription kit (Qiagen, Cat: 330411, Germany) starting from 1 μg of the treated RNA according to the manufacturer’s instructions. One microgram of each cDNA was used as a template for amplification using SYBR Green Master Mix (Qiagen, Cat: 330500, Germany) and gene-specific primers. Real-time PCR primers (GAPDH, caspase 3, iNOS, CYP1A and MT1) were designed based on the sequences of Common Carp (*Cyprinus Carpio*) using the Oligo 6.0 and Primer 5.0 design programs. Primer sequences and the corresponding PCR conditions are listed in Table 2. GAPDH was used as the RT-qPCR internal control. Each PCR reaction was performed in triplicate. The specificity of PCR amplification was confirmed by agarose gel electrophoresis and melting curve analysis. Relative gene expression was determined with the 2^−ΔΔCT^ method [45].

**Table 2:** Primer sequences and RT-qPCR conditions.

Primer	Sequences (5′-3′)	Length (bp)	Accession no	Reaction Conditions
GAPDH	F: TCCGTCTTGAGAAACCTGTG	212	AJ870982.1	94 °C 15 s/55 °C 30 s/72 °C 30 s (40 cycles)
	R: ATTCACCTACACTGCACCCA			
iNOS	F: GTTGGTACATGGGCACTGAG	201	AJ242906.1	94 °C 15 s/58 °C 30 s/72 °C 30 s (40 cycles)
	R: TATGGTGGTCGGTAATGGTG			
Caspase 3	F: TTGCTTCTCACTGGCTCATC	166	KF055462.1	94 °C 15 s/57 °C 30 s/72 °C 30 s (40 cycles)
	R: CTCCAAACGTTTAACGAGCA			
CYP1A	F: TTCCTTCCTTTCACCATTCC	149	AB048939.1	94 °C 15 s/59 °C 30 s/72 °C 30 s (40 cycle)
	R: TCCAGATTGAAGCTTGATGG			
MT1	F: TGCGCCAAGAGTAAGTGTTT	156	AY789469.1	94 °C 15 s/60 °C 30 s/72 °C 30 s (40 cycles)
	R: ACCACAATTGCAAGTTCCAG			

### 2.6 Statistical analysis

One-way ANOVA followed by Tukey’s post hoc test was used to analyze the mRNA levels of caspase 3, iNOS, CYP1A and MT1 in treated and control groups. The analysis was performed using the GraphPad Prism software version 7.0 (Version 7.0, California, USA). RT-qPCR results were expressed as the mean ± SEM (standard error of the mean). Differences were considered to be statistically significant with p values of p < 0.05, *p <* 0.01 and *p <* 0.001. For the histopathological scores, obtained scores were analyzed statistically by using SPSS statistical software (SPSS for windows, version 20.0). Differences in measured parameters among the groups were analyzed with a nonparametric test (Kruskal-Wallis). Dual comparisons between groups exhibiting significant values were evaluated with a Mann-Whitney *U* test (*p <* 0.05).

## 3 Results

### 3.1 Histopathological changes

**Fig. 2:**
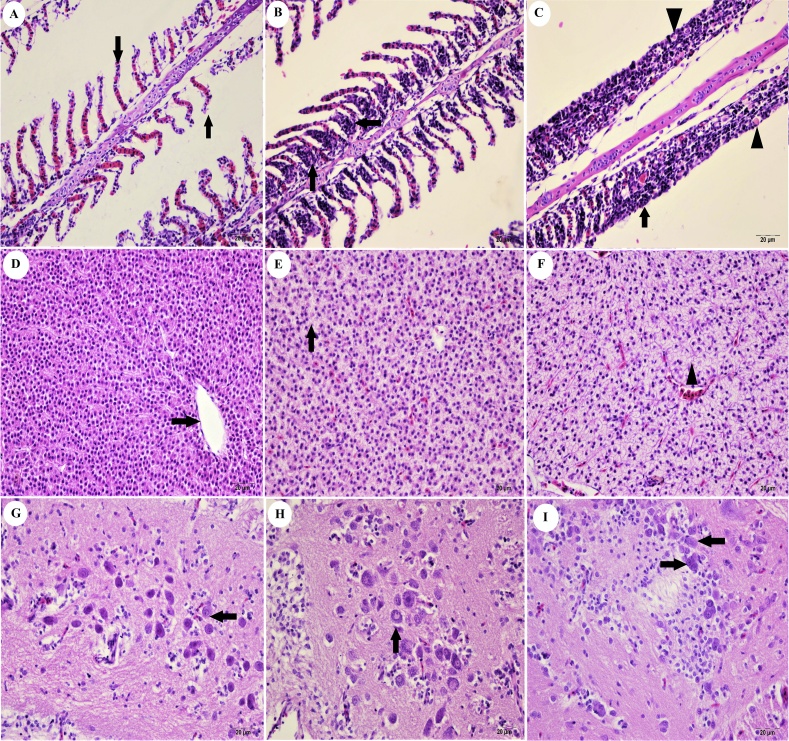
Histopathological changes of imidacloprid toxication (96 h). A) Normal histology of the gill structure (arrows). Control group. H&E. 20 μm. B) Hyperplasia of gill epithelium. Low dose toxic group, gill tissue. H&E. 20 μm. C) Hyperplasia of gill epithelium (arrow) and mucous cell hyperplasia (arrow head). High dose toxic group, gill tissue. H&E. 20 μm. D) Normal histology of liver, vena centralis (arrow). Control group. H&E. 20 μm. E). Hydropic and vacuolar degeneration in hepatocytes (arrow). Low dose toxic group, liver tissue. H&E. 20 μm. F). Diffus hydropic degeneration and focal necrosis (arrow head) in hepatocytes. High dose toxic group, liver tissue. H&E. 20 μm. G) Normal histology of neurons (arrows) in optic lobe. Control group. H&E. 20 μm. H). Normal histology of neurons (arrows) in optic lobe. Low dose toxic group. H&E. 20 μm. I), Degenerated neurons (arrows) in optic lobe. High dose toxic group H&E. 20 μm.

Tissues obtained from the control groups showed normal features (Fig. 2A,D,G). No apparent lesions were detected in the histopathological examination of brain tissue, apart from some neurodegeneration in samples exposed to the high dose of IMI (280 mg/L) for 96 h. Hyperplasia of secondary lamellar cells (Fig. 2B) and mucous cell hyperplasia in gills (Fig. 2C), as well as hydropic degeneration in hepatocytes (Fig. 2E) and necrosis in liver (Fig. 2F), were observed in the evaluation of samples exposed to 140 and 280 mg/L of imidacloprid for 96 h. The severity of the histopathological lesions that were detected in all groups increase proportionally with toxic dose right and exposure time. There was statistically significant differences between application groups (*p <* 0.05). The most obvious lesions in liver and gills at both doses were detected at the 96 h exposure.

### 3.2 Immunofluorescence assessments

**Fig. 3:**
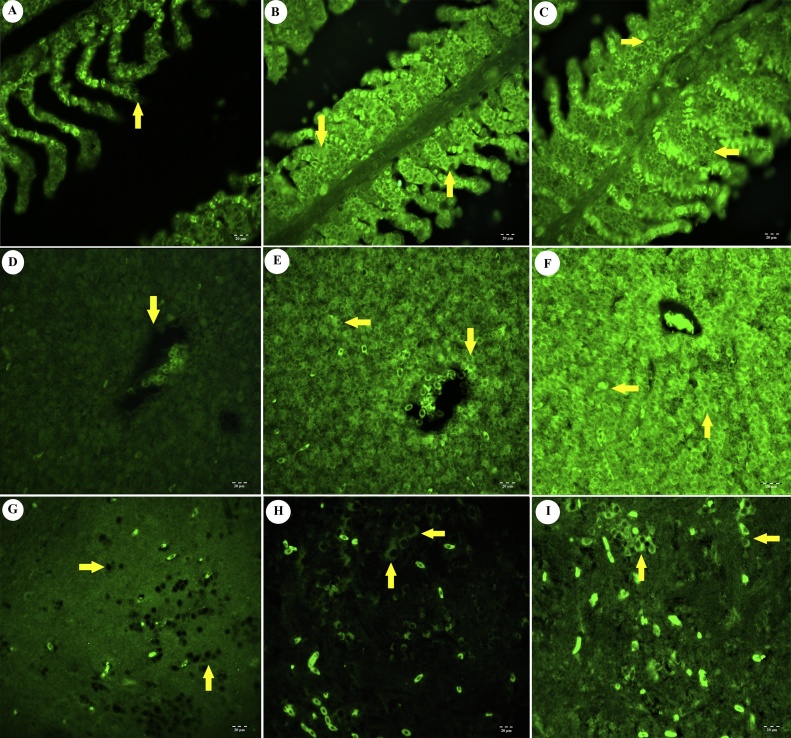
Immunoflorescence staining results of 8-OHdG (96 h). A) Lamella of gill tissue, (arrow). Control group. IF. 20 μm. B) Positivity in lamellar cells of gill tissue (arrows). Low dose toxic group. IF. 20 μm. C) Positivity in lamellar cells of gill tissue (arrows). High dose toxic group. IF. 20 μm. D) Liver tissue, vena centralis (arrow). Control group. IF. 20 μm. E) Positive reactions in hepatocytes (arrows). Low dose toxic group. IF. 20 μm. F) Positive reactions in hepatocytes (arrows). High dose toxic group. IF. 20 μm. G) Neurons (arrows). Control group. IF. 20 μm. H) Positivity in neurons (arrows). Low dose toxic group. IF. 20 μm. I) Positivity in neurons (arrows). High dose toxic group. IF. 20 μm.

**Fig. 4:**
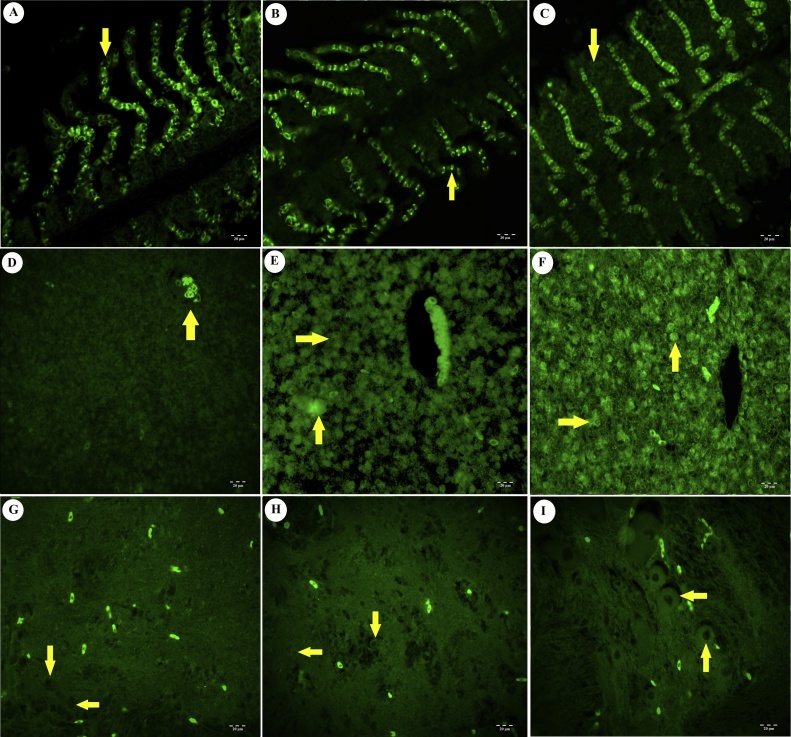
Immunoflorescence staining results of iNOS (96 h). A) Lamella of gill tissue (arrow). Control group. IF. 20 μm. B) Weak positive reaction in lamellar cells of gill tissue (arrows). Low dose toxic group. IF. 20 μm. C) Positivity in lamellar cells of gill tissue (arrow). High dose toxic group. IF. 20 μm. D) Liver tissue, vena centralis (arrow). Control group. IF. 20 μm. E) Positive reactions in hepatocytes (arrows). Low dose toxic group. IF. 20 μm. F) Positive reactions in hepatocytes (arrows). High dose toxic group. IF. 20 μm. G) Weak positive reactions in neurons (arrows). Control group. IF. 20 μm. H) Positivity in neurons (arrows). Low dose toxic group. IF. 20 μm. I) Positivity in neurons (arrows). High dose toxic group. IF. 20 μm.

**Fig. 5:**
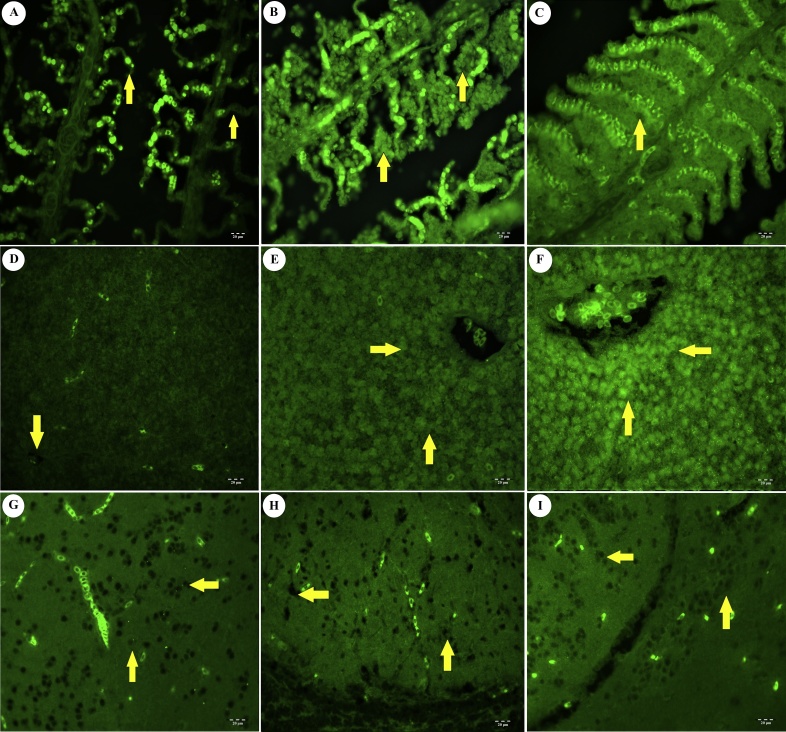
Immunoflorescence staining results of TNF-α (96 h). A). Lamella of gill tissue (arrow). Control group. IF. 20 μm. B) Positive reaction in lamellar cells of gill tissue (arrows). Low dose toxic group. IF. 20 μm. C) Positivity in lamellar cells of gill tissue (arrow). High dose toxic group. IF. 20 μm. D) Liver tissue, vena centralis (arrow). Control group. IF. 20 μm. E) Positive reactions in hepatocytes (arrows). Low dose toxic group. IF. 20 μm. F) Positive reactions in hepatocytes (arrows). High dose toxic group. IF. 20 μm. G) Weak positive reactions in neurons (arrows). Control group. IF. 20 μm. H) Weak positivity in neurons (arrows). Low dose toxic group. IF. 20 μm. I) Positivity in neurons (arrows). High dose toxic group. IF. 20 μm.

Weak positive signals were observed for all the biomarkers in tissues obtained from the control groups (Figs. 3A, D, G, 4A, D, G, 5A, D, G ) and 24 h application groups. There was no statistically significant differences between these groups (*p >* 0.05). Positive signals for 8-OHdG were observed in gill epithelial cells (Fig. 3B), hepatocytes in the liver (Fig. 3E) and neurons in the brain (Fig. 3H) in the groups exposed to 140 mg/L IMI. Strong positive signals were detected in all tissues (Fig 3C, F, I) in the groups treated with 280 mg/L. Positive immunofluorescence signals for iNOS were not clearly detected in gill tissue obtained from the treated groups (Fig. 4B, C) (*p >* *0,05*). However, iNOS positivity was observed in hepatocytes (Fig. 4E, F) and neurons (Fig. 4H, I). Positive signals for TNF-α were detected in gills (Fig. 5B, C) and liver (Fig. 5E, F). In addition, a weak, positive signal for TNF-α was observed in the brain (Fig. 5H, I). The positive immunofluorescence signal for 8-OHdG was significantly stronger than those of the other biomarkers in tissues obtained from the treated groups (Fig. 3) (*p <* 0.05). Lastly, 2.5D immunopositivities for TNF-α, iNOS and 8-OHdG were observed, as shown in Fig. S1B. C. D)

### 3.3 The effect of imidacloprid on caspase 3, iNOS, CYP1A and MT1 gene expression

**Fig. 6:**
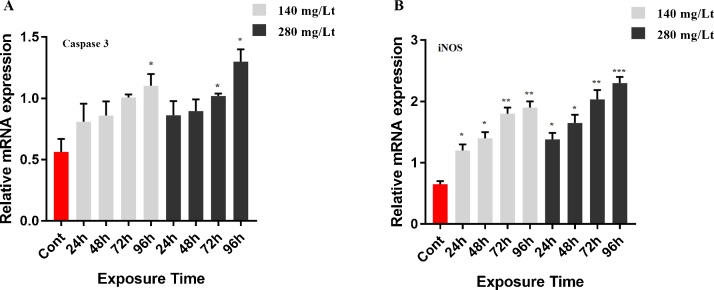
Effect of 140 mg/L and 280 mg/L doses of IMI exposure on mRNA transcript levels of caspase-3 and iNOS in the brain of Common Carp. Values represent the mean ± SD of 3 indipendent samples; error bars indicate standard deviation. Statistical significance (* P ˂ 0.05, ** p < 0.01, *** p < 0.001) was analyzed using a factorial ANOVA. A) Represent the relative mRNA expression levels of caspase-3. B) Represent the relative mRNA expression levels of iNOS.

**Fig. 7:**
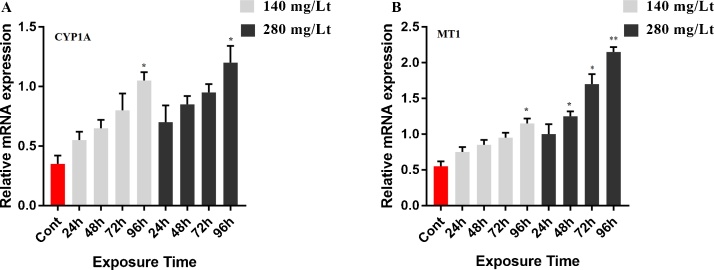
Effect of 140 mg/L and 280 mg/L doses of IMI exposure on mRNA transcript levels of CYP1A and MT1 in the brain of Common Carp. Values represent the mean ± SD of 3 indipendent samples; error bars indicate standard deviation. Statistical significance (* P ˂ 0.05, ** p < 0.01) was analyzed using a factorial ANOVA. A) Represent the relative mRNA expression levels of CYP1A. B) Represent the relative mRNA expression levels of MT1.

Caspase 3, iNOS, CYP1A and MT1 mRNA levels were measured by RT-qPCR. The mRNA level of caspase 3 in brain tissue was not significantly up-regulated when using both the low dose (140 mg/L for 24 h, 48 h, 72 h) and the high dose (280 mg/L for 24 h, 48 h,) of IMI (*p >* 0.05). However, when treating with the low dose of IMI for 96 h and the high dose of IMI for 96 h, 72 h, the caspase 3 gene expression was up-regulated compared to that of the control group (*p <* 0.05; Fig. 6A). Quantitative mRNA analyses have indicated that iNOS mRNA was up-regulated in tissue exposed to IMI (both 140 mg/L and 280 mg/L doses) compared to the control group (*p < *0.05, *p < *0.01 and *p < *0.001; Fig. 6B). Furthermore, neither the high nor the low IMI dose for 24 h, 48 h and 72 h caused the up-regulation of the CYP1A gene in the brain (*p > 0.05*). However, 96 h IMI exposure with the low and high dose was significantly up-regulated CYP1A gene expression (Fig. 7A). Similarly, expression of the MT1 gene was not up-regulated by exposure to the low dose of IMI except 96 h (Fig. 7B). However, exposure to a high dose of IMI for 48 h, 72 h and 96 h induced up-regulation of the MT1 gene in treated tissues compared to the control group (*p <* 0.05 and *p <* 0.01; Fig. 7B). Gene expression data normality and homogenity for caspase 3, iNOS, CYP1A and MT1 were shown in Table S1. The melting curve peaks of caspase 3, iNOS, CYP1A and MT1 gene expression are shown in Fig. S2.

## 4 Discussion

Pesticides have been consistently used to eliminate insects and weeds, but their uncontrolled use has resulted in environmental contamination leading to harmful effects on non-target organisms such as humans and aquatic animals [46]. Pesticides induce inflammation, oxidative stress, and mitochondrial dysfunction in vertebrates [17,47]. Although imidacloprid has been widely used in recent times, data on its potential toxic effects on mammals or aquatic animals such as fish are insufficient. Imidacloprid is a neonicotinoid insecticide that causes paralysis of the central nervous system in insects [48,49]. In addition, it may affect the central nervous system of humans and animals.

Imidacloprid has been shown to induce histopathological lesions, including hydropic degeneration, pyknotic nuclei, and loss of hepatocytes, in *Oreochromis niloticus* [50]. Similarly, IMI treatment can cause central vein dilation and sinusoids between hepatocytes and hepatotoxicity and nephrotoxicity in albino rats [14,51]. Bhardwaj et al. (2010) reported that mild pathological changes occurred as a consequence of oral toxicity of IMI in female rats [52]. Previous studies have shown that IMI exposure leads to histopathological changes and genotoxic effects in the non-target organisms such as fish and rabbit [10,50,53]. In addition, long-term exposure of IMI can cause oxidative stress in rabbits [12]. However, currently, there is limited information available regarding the histopathological effects of IMI on the gills and brain of fish because researchers have not clearly evaluated these effects. In the present study, we observed hyperplasia of secondary lamellar cells and mucous cell hyperplasia in gills, as well as hydropic degeneration in hepatocytes and necrosis in liver. However, severe histopathological lesions were not detected in brain tissue, apart from mild neurodegeneration. These results indicated that acute IMI toxicity primarily affected the gills and liver.

An immunofluorescence assay (IF) was performed to assess the activation of iNOS, 8-OHdG and TNF-α in IMI-exposed gills, liver and brain of the common carp. IF is more sensitive than immunohistochemistry (IHC) and histopathological examination [54]. In the current study, severe 8-OHdG immunopositivity was observed in the gills, liver and brain, whereas iNOS positivity was observed especially in the liver and brain, and TNF-α positivity was observed in the gills and liver. Therefore, our IF results indicated that acute IMI toxicity induced inflammation and oxidative stress especially in the gills and liver.

In the present study, caspase 3 mRNA expression was not significantly up-regulated in a time-dependent manner in the brain of the imidacloprid-exposed common carp; only the exposure to the low and high dose of imidacloprid for 96 h promoted caspase 3 expression (*p <* 0.05). Previous studies have shown that imidacloprid intoxication induces the caspase 3-dependent apoptosis pathway in the brain of honey bees and neonatal rats [33]. IMI treatment also leads to neurobehavioral impairments, oxidative stress and DNA damage in zebrafish (*Danio rerio*) [55]. Mesnage et al. (2014) showed that imidacloprid increases the caspase 3 mRNA expression level in human cell lines (HepG2, HEK293, and JEG3) [56]. Furthermore, imidacloprid exposure results in apoptosis in chicken embryos [57]. There are currently no reports concerning caspase 3 activation after IMI treatment in the brain of the common carp. Our results on gene transcription indicated that acute IMI toxicity moderately induce apoptosis in the brain of common carp, since IMI could be insufficiently accumulated in the brain. However, chronic IMI toxicity may lead to apoptosis in the brain of the common carp.

Duzguner and Erdogan (2012) observed that iNOS synthesis was increased in the liver and central nervous system of rats exposed to IMI and that IMI toxicity induced oxidative stress and inflammation in rat livers and brains [17]. Lanore et al. (2015) reported that imidacloprid intoxication caused oxidative stress and DNA damage in the reproductive organs of Wistar rats [58]. A previous study indicated that oral administration of imidacloprid induced oxidative stress in rats [59]. A study by Aydin (2011) demonstrated that thiacloprid, which is another neonicotinoid insecticide, activates iNOS in the lymphoid organs of rats [60]. Several studies on imidacloprid toxicity have focused on rats, whereas there is insufficient information on iNOS activation in fish owing to the scarcity of reports on imidacloprid toxicity in aquatic animals. In the present study, iNOS mRNA expression was significantly increased in the brains of imidacloprid-exposed common carp (*p <* 0.05, *p <* 0.01 and *p <* 0.001). This result confirmed the immunopositivity of iNOS and indicated that IMI exposure triggers oxidative stress and inflammation in the brain tissue.

Cytochrome P450 (CYP) enzymes are important modulators of chemical detoxification of herbicides, pesticides, and carcinogens in animals [61,62]. In addition, CYP1A, a member of the cytochrome P450 (CYP) enzyme superfamily, is considered to be an important enzyme contributing to drug and chemical metabolism in fish and other animals [63]. It may provide useful information about fish behavior and health resulting from environmental stress, pesticide intoxication and water pollution [61]. Chlorpyrifos (CPF) exposure down-regulates CYP3A gene expression in the liver of goldfish [64]. Furthermore, a previous study showed that chlorpyrifos (CPF) and atrazine (ATR) did not change the expression of CYP1A in the common carp gills [65]. Dioxin-like PCB126 is an organic pollutant that significantly up-regulates CYP1A mRNA expression in zebrafish (*Danio rerio*) embryos [57]. Li et al. (2016) showed that the biocide tributyltin (TBT) induced the expression of cytochrome P450 1 (CYP450 1) in the liver, gills and muscle of the common carp [66]. In this study, we demonstrated that CYP1A expression was not changed (*p >* 0.05) except with the low and high doses of IMI for 96 h in the brain (*p <* 0.05). Therefore, acute IMI toxicity may not significantly affect CYP1A expression in the brain of the common carp.

MTs play a role in the regulation and detoxification of metals. In addition, several other factors such as antibiotic use, freezing, anoxia and pesticide contamination can lead to the induction of MTs [67,68]. Induction levels of MT1 can be used to evaluate the pollution degree in water [69]. Previous studies have shown that pesticide toxicity leads to oxidative stress in animals [70,71]. It is known that MT1 decreases the harmful effects of free radicals and reactive oxygen species (ROS) produced by oxidative stress. In the present study, MT1 gene expression was significantly up-regulated in the brains exposed to the low dose of IMI for 96 h and the high dose of IMI for 72 h and 96 h (*p <* 0.05 and *p <* 0.01), whereas the other doses and exposure time points did not induce any change. Therefore, acute IMI toxicity does not promote the induction of MT1. The up-regulation of MT1 in the brain exposed to a high dose of IMI for 72 h and 96 h indicates that IMI toxicity leads to oxidative stress at these time points. In addition, chronic IMI toxicity may induce oxidative stress-dependent MT1 synthesis in the brain of the common carp. In the future, it will be necessary to investigate the MT1 response to chronic IMI toxicity in the common carp.

In conclusion, acute IMI toxicity caused histopathological lesions in the gills and liver; activation of 8-OHdG, iNOS and TNF-α in the gills, liver and brain; significant up-regulation of the iNOS gene; and a modest up-regulation of the caspase 3, CYP1A and MT1 genes in the brain. Based on these results, we concluded that inflammation and oxidative stress are induced by IMI exposure in the common carp. Therefore, the assessment of fish mortality in aquatic ecosystems will be facilitated by histopathological examinations. The use of pesticides should not be left to the initiative of farmers. Furthermore, it should not be forgotten that all pesticides have been tested and evaluated because of their risk of synergistically cause further and permanent damage; for this reason, a pesticide atlas should be developed for aquatic animals.

## References

[1] Elbert A., Haas M., Springer B., Thielert W., Nauen R. (2008). Applied aspects of neonicotinoid uses in crop protection. Pest Manag. Sci..

[2] Jeschke P., Nauen R., Schindled M., Elbert A. (2011). Overview of the statusand global strategy for neonicotinoids. J. Agric. Food. Chem..

[3] Matsuda K., Shimomura M., Ihara M., Akamatsu M., Sattelle D.B. (2005). Neonicotinoids show selective and diverse actions on their nicotinic receptor targets: electrophysiology, molecular biology, and receptor modeling studies. Biosci. Biotechnol. Biochem..

[4] Dryden M.W., Denenberg T.M., Bunch S. (2000). Control of fleas on naturally infested dogs and cats and in private residences with topical spot applications of fipronil or imidacloprid. Vet. Parasitol..

[5] Weston D.P., Chen D., Lydy M.J. (2015). Stormwater-related transport of the insecticides bifenthrin, fipronil, imidacloprid, and chlorpyrifos into a tidal wetland, San Francisco Bay, California. Sci. Total Environ..

[6] Gupta S., Gajbhiye V.T., Kalpana, Agnihotri N.P. (2002). Leaching behavior of imidacloprid formulations in soil. Bull. Environ. Contam. Toxicol..

[7] Armbrust K.L., Peeler H.B. (2002). Effects of formulation on the run-off of imidacloprid from turf. Pest Manage Sci..

[8] Tišler T., Jemec A., Mozetic B., Trebše T. (2009). Hazard identification of imidacloprid to aquatic environment. Chemosphere.

[9] Karabay N.U., Oguz M.G. (2005). Cytogenetic and genotoxic effects of the insecticides, imidacloprid and methamidophos. Genet. Mol. Res..

[10] Stivaktakis P.D., Kavvalakis M.P., Tzatzarakis M.N., Alegakis A.K., Panagiotakis M.N., Fragkiadaki P., Vakonaki E., Ozcagli E., Hayes W.A., Rakitskii V.N., Tsatsakis A.M. (2016). Long-term exposure of rabbits to imidaclorpid as quantified in blood induces genotoxic effect. Chemosphere.

[11] Vardavas A.I., Ozcagli E., Fragkiadaki P., Stivaktakis P.D., Tzatzarakis M.N., Kaloudis K., Tsardi M., Datseri G., Tsiaoussis J., Tsitsimpikou C., Carvalho F., Tsatsakis A.M. (2016).

[12] Stivaktakis P., Kavvalakis M., Goutzourelas N., Stagos D., Tzatzarakis M., Kyriakakis M., Rezaee R., Kouretas D., Hayes W., Tsatsakis A. (2014). Evaluation of oxidative stress in long-term exposed rabbits to subtoxic levels of imidacloprid. Toxicol. Lett..

[13] Kavvalakis M.N., Tzatzarakis E.P., Theodoropoulou E.G., Barbounis A.K. (2013). Development and application of LC–APCI–MS method for biomonitoring of animal and human exposure to imidacloprid. Chemosphere.

[14] Arfat Y., Mahmood N., Tahir M.U., Rashid M., Anjum S., Zhao F., Li D.J., Sun Y.L., Hu L., Zhihao C., Yin C., Shang P., Qian A.R. (2014). Effect of imidacloprid on hepatotoxicity and nephrotoxicity in male albino mice. Toxicol. Rep..

[15] Shukla S., Jhamtani R.C., Dahiya M.S., Agarwala R. (2014). Oxidative injury caused by individual and combined exposure of neonicotinoid, organophosphate and herbicide in zebrafish. Toxicol. Rep..

[16] Mohamed A.A.R., Mohamed W.A.M., Khater S.I. (2017). Imidacloprid induces various toxicological effects related to the expression of 3b-HSD, NR5A1, and OGG1 genes in mature and immature rats. Environ. Pollut..

[17] Duzguner V., Erdogan S. (2012). Chronic exposure to imidacloprid induces inflammation and oxidative stress in the liver & central nervous system of rats. Pestic. Biochem. Physiol..

[18] Knowles R.G. (1996). Nitric oxide synthases. Biochem. Soc. Trans..

[19] Muniz J.F., McCauley L., Scherer J., Lasarev M., Koshy M., Kow Y.W., NazarStewart V., Kisby G.E. (2008). Biomarkers of oxidative stress and DNA damage in agricultural workers: a pilot study. Toxicol. Appl. Pharmacol..

[20] Arora D., Siddiqui M.H., Sharma P.K., Shukla Y. (2016). Deltamethrin induced RIPK3-mediated caspase-independent non-apoptotic cell death in rat primary hepatocytes. Biochem. Biophys. Res. Commun..

[21] Villaño D., Vilaplana C., Medina S., Cejuela-Anta R., Martínez-Sanz J.M., Gil P., Genieser H.G., Ferreres F., Gil-Izquierdo A. (2015). Effect of elite physical exercise by triathletes on seven catabolites of DNA oxidation. Free Radic. Res..

[22] Arslan H., Altun S., Özdemir S. (2017). Acute toxication of deltamethrin results in activation of iNOS, 8-OHdG and up-regulation of caspase 3, iNOS gene expression in common carp (*Cyprinus carpio* L.). Aquat. Toxicol..

[23] Arslan H., Özdemir S., Altun S. (2017). Cypermethrin toxication leads to histopathological lesions and induces inflammation and apoptosis in common carp (*Cyprinus carpio* L.). Chemosphere.

[24] Alves J.C., Andressa F., Fabricio O., Souto F. (2009). Regulation of chemokine receptor by toll-like receptor 2 is critical to neutrophil migration and resistance to polymicrobial sepsis. Proc. Natl. Acad. Sci. U. S. A..

[25] Singh A.K., Jiang Y. (2003). Lipopolysaccharide (LPS) induced activation of the immune system in control rats and rats chronically exposed to a low level of the organothiophosphate insecticide, acephate. Toxicol. Ind. Health..

[26] Pandit S., Ramneek B., Singh, Sethi R.S. (2016). Imidacloprid induced histomorphological changes and expression of TLR-4 and TNFα in lung. Pestic. Biochem. Physiol..

[27] Ojha A., Gupta Y.K. (2016). Study of commonly used organophosphate pesticides that induced oxidative stress and apoptosis in peripheral blood lymphocytes of rats. Hum. Exp. Toxicol..

[28] Yang Y., Zong M., Xu W., Zhang Y., Wang B., Yang M., Tao L. (2017). Natural pyrethrins induces apoptosis in human hepatocyte cells via Bax- and Bcl-2-mediated mitochondrial pathway. Chem. Biol. Interact..

[29] Deponte M., Becker K. (2004). Plasmodium falciparum-do killers commit suicide. Trends Parasitol..

[30] Wong R.S.Y. (2011). Apoptosis in cancer: from pathogenesis to treatment. J. Exp. Clin. Cancer Res..

[31] Elmore S. (2007). Apoptosis a review of programmed cell death. Toxic. Pat..

[32] Xu W.N., Liu W.B., Liu Z.P. (2009). Trichlorfon-induced apoptosis in hepatocyte primary cultures of Carassius auratus gibelio. Chemosphere.

[33] Wu Y.Y., Zhou T., Wang Q., Dai P.L., Xu S.F., Jia H.R., Wang X. (2015). Programmed cell death in the honey bee (Apis mellifera) (Hymenoptera: apidae) worker brain induced by imidacloprid. J. Econ. Entomol..

[34] Kagi J.H.R., Schaffer A. (1988). Biochemistry of metallothionein. Biochemistry.

[35] Ali K.S., Ferencz Á., Deér A.K., Nemcsók J., Hermesz E. (2009). Expression of two metallothionein genes in different brain regions of common carp. Acta Biol. Hung..

[36] Ferencz Á., Hermesz E. (2015). Impact of acute Cd^2^⁺ exposure on the antioxidant defence systems in the skin and red blood cells of common carp (*Cyprinus carpio*). Environ. Sci. Pollut. Res. Int..

[37] Pelkonen O., Mäenpää J., Taavitsainen P., Rautio A., Raunio H. (1998). Inhibition and induction of human cytochrome P450 (CYP) enzymes. Xenobiotica.

[38] Linde-Arias A.R., Inácio A.F., Novo L.A., de Alburquerque C., Moreira J.C. (2008). Multibiomarker approach in fish to assess the impact of pollution in a large Brazilian river. Paraiba do Sul. Environ. Pollut..

[39] Pelkonen O., Breimer D.D., Welling P.G., Balant L.P. (1994). Role of environmental factors in the pharmacokinetics of drugs: considerations with respect to animal models, P-450 enzymes, and probe drugs.

[40] Oliva M., Gravato C., Guilhermino L. (2014). et al., EROD activity and cytochrome P4501A induction in liver and gills of Senegal *Solea solea* senegalensis from a polluted Huelva Estuary(SW Spain). Comp. Biochem. Physiol. Toxicol. Pharmacol..

[41] Beijer K., Gao K., Jonsson M.E. (2013). Effluent from drug manufacturing affects cytochrome P450 1 regulation and function in fish. Chemosphere.

[42] Erdoğan O., Ceyhun S.B., Ekinci D., Aksakal E. (2011). Impact of deltamethrin exposure on mRNA expression levels of metallothionein A, B and cytochrome P450 1A in rainbow trout muscles. Gene. Sep..

[43] (2017). International Union of Pure and Applied Chemistry. PPDB: PesticidesProperties DataBase. http://sitem.herts.ac.uk/aeru/iupac/atoz.htm.

[44] Qadir S., Iqbal F. (2016). Effect of subleathal concentrtion of imidacloprid on the histology of heart, liver and kidney in Labeo rohitr. Pak. J. Pharm. Sci..

[45] Livak K.J., Schmittgen T.D. (2001). Analysis of relative gene expression data using real-time quantitative PCR and the 2 (-Delta Delta C(T)) Method. Methods.

[46] Qian H., Chen W., Sun L., Jin Y., Liu W., Fu Z. (2009). Inhibitory effects of paraquat on photosynthesis and the responseto oxidative stress in *Chlorella vulgaris*. Ecotoxicology.

[47] Ascherio H., Chen M.G., Weisskopf E., O’Reilly M.L., McCullough E.E., Calle M.A., Schwarzschild, Thun M.J. (2006). Pesticide exposure and risk for Parkinson’s disease. Ann. Neurol.

[48] Raymond-Delpech V., Matsuda K., Sattelle B.M., Rauh J.J., Sattelle D.B. (2005). Ion channels: molecular targets of neuroactive insecticides. Invert. Neurosci..

[49] Tomizawa M., Casida J.E. (2005). Neonicotinoid insecticide toxicology: mechanisms of selective action. Annu. Rev. Pharmacol. Toxicol..

[50] Ansoar-Rodríguez Y., Christofoletti C.A., Correia J.E., de Souza R.B., Moreira-de-Sousa C., Marcato A.C., Bueno O.C., Malaspina O., Silva-Zacarin E.C., Fontanetti C.S. (2016). Liver alterations in *Oreochromis niloticus* (Pisces) induced by insecticide imidacloprid: histopathology and heat shock protein in situ localization. J. Environ. Sci. Health B.

[51] Toor H.K., Sangha G.K., Khera K.S. (2013). Imidacloprid induced histological and biochemical alterations in liver of female albino rats. Pestic. Biochem. Physiol..

[52] Bhardwaj S., Srivastava M.K., Kapoor U., Srivastava L.P. (2010). A 90 days oral toxicity of imidacloprid in female rats: morphological, biochemical and histopathological evaluations. Food Chem. Toxicol..

[53] Vohra P., Khera K.S., Sangha G.K. (2014). Physiological, biochemical and histological alterations induced by administration of imidacloprid in female albino rats. Pestic. Biochem. Physiol..

[54] Giltnane J.M., Molinaro A., Cheng H., Robinson A., Turbin D., Gelmon K., Huntsman D., Rimm D.L. (2008). Comparison of quantitative immunofluorescence with conventional methods for HER2/neu testing with respect to response to trastuzumab therapy in metastatic breast cancer. Arch. Pathol. Lab. Med..

[55] Emily B., Crosby J., Bailey M., Anthony N.O., Levin E.D. (2015). Neurobehavioral impairments caused by developmental imidacloprid exposure in zebrafish. Neurotoxicol. Teratol..

[56] Mesnage R., Defarge N., Spiroux de Vendômois J., Séralini G.E. (2014). Major pesticides are more toxic to human cells than their declared active principles. BioMed Res. Int..

[57] Liu H., Nie F.H., Lin H.Y., Ma Y., Ju X.H., Chen J.J., Gooneratne R. (2016). Developmental toxicity EROD, and CYP1A mRNA expression in zebrafish embryos exposed to dioxin-like PCB126. Environ. Toxicol..

[58] Lonare M., Kumar M., Raut S., More A., Doltade S., Badgujar P., Telang A. (2016). Evaluation of ameliorative effect of curcumin on imidacloprid-induced male reproductive toxicity in wistar rats. Environ. Toxicol..

[59] Soujanya S., Lakshman M., Kumar A.A., Reddy A.G. (2013). Evaluation of the protective role of vitamin C in imidacloprid-induced hepatotoxicity in male Albino rats. J. Nat. Sci. Biol. Med..

[60] Aydin B. (2011). Effects of thiacloprid deltamethrin and their combination on oxidative stress in lymphoid organs, polymorphonuclear leukocytes and plasma of rats. Pest. Biochem. Physiol..

[61] Uno T., Ishizuka M., Itakur T. (2012). Cytochrome P450 (CYP) in fish. Environ. Toxicol. Pharmacol..

[62] Nelson D.R. (2009). The cytochrome P450 homepage. Hum. Genomics.

[63] Tsuchiya Y., Nakajima M., Yokoi T. (2005). Cytochrome P450-mediated metabolism of estrogens and its regulation inhuman. Cancer Lett..

[64] Ma J., Liu Y., Niu D., Li X. (2015). Effects of chlorpyrifos on the transcription of CYP3A cDNA, activity of acetylcholinesterase, and oxidative stress response of goldfish (*Carassius auratus*). Environ. Toxicol..

[65] Fu Y., Li M., Liu C., Qu J.P., Zhu W.J., Xing H.J., Xu S.W., Li S. (2013). Effect of atrazine and chlorpyrifos exposure on cytochrome P450 contents and enzyme activities in common carp gills. Ecotoxicol. Environ. Saf..

[66] Li Z.H., Zhong L.Q., Mu W.N., Wu Y.H. (2016). Effects of chronic exposure to tributyltin on tissue-specific cytochrome P450 1 regulation in juvenile common carp. Xenobiotica.

[67] Mosleh Y.Y., Paris-Palacios S., Arnoult F., Couderchet M., Biagianti-Risbourg S., Vernet G. (2014). Metallothionein induction in aquatic oligochaete *Tubifex tubifex* exposed to herbicide isoproturon. Environ. Toxicol..

[68] English T.E., Storey K.B. (2003). Freezing and anoxia stresses induce expression of metallothionein in the foot muscle and hepatopancreas of the marine gastropod *Littorina littorea*. J. Exp. Biol..

[69] Roch M., McCarter J.A., Matheson A.T., Clark M.J.R., Olafson R.W. (1982). Hepatic metallothionein in rainbow trout as an indicator of metal pollution in the Campbell River system. Can. J. Fish. Aquat. Sci..

[70] Mena S., Ortega A., Estrela J.M. (2009). Mini review: oxidative stress in environmental-induced carcinogenesis. Mutat. Res..

[71] Nasuti C., Gabbianelli R., Falcioni M.L., Di Stefano A., Sozio P., Cantalamessa F. (2007). Dopaminergic system modulation, behavioral changes, and oxidative stress after neonatal administration of pyrethroids. Toxicology.

