# Supplementary material for: Imidacloprid exposure cause the histopathological changes, activation of TNF-α, iNOS, 8-OHdG biomarkers, and alteration of caspase 3, iNOS, CYP1A, MT1 gene expression levels in common carp (Cyprinus carpio L.)
Source: Toxicol Rep. 2017 Dec 27;5:125–33. doi: 10.1016/j.toxrep.2017.12.019 (PMC5751999; doi:10.1016/j.toxrep.2017.12.019)

**S1: 2.5D immunopositivies for TNF-α, iNOS and 8-OHdG.** A) Control group. B) TNF- α activation. C) iNOS activation. D) 8-OHdG activation.


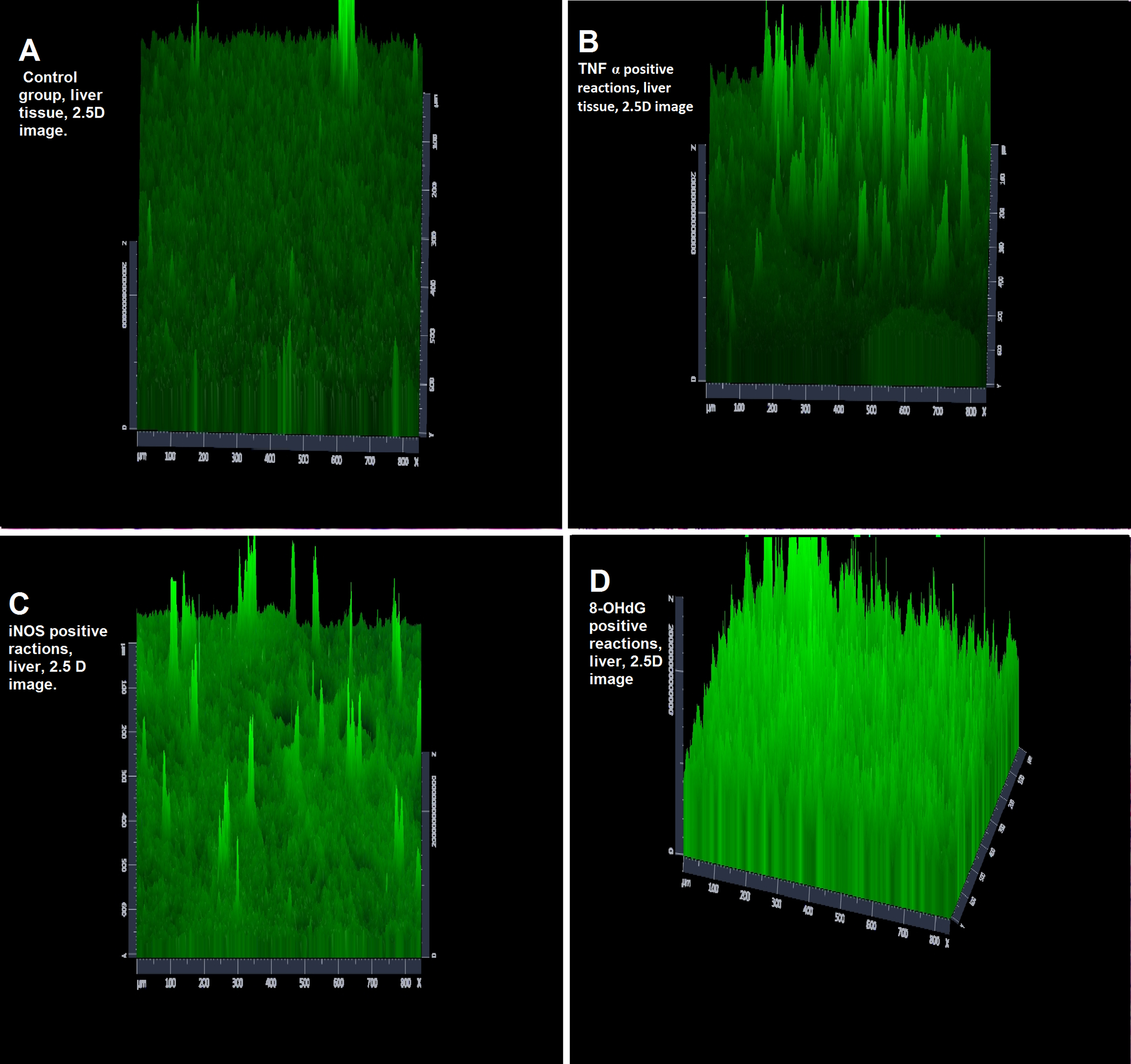


**Figure S2: Melting curve analysis of caspase 3, iNOS, CYP1A and MT1 genes.**


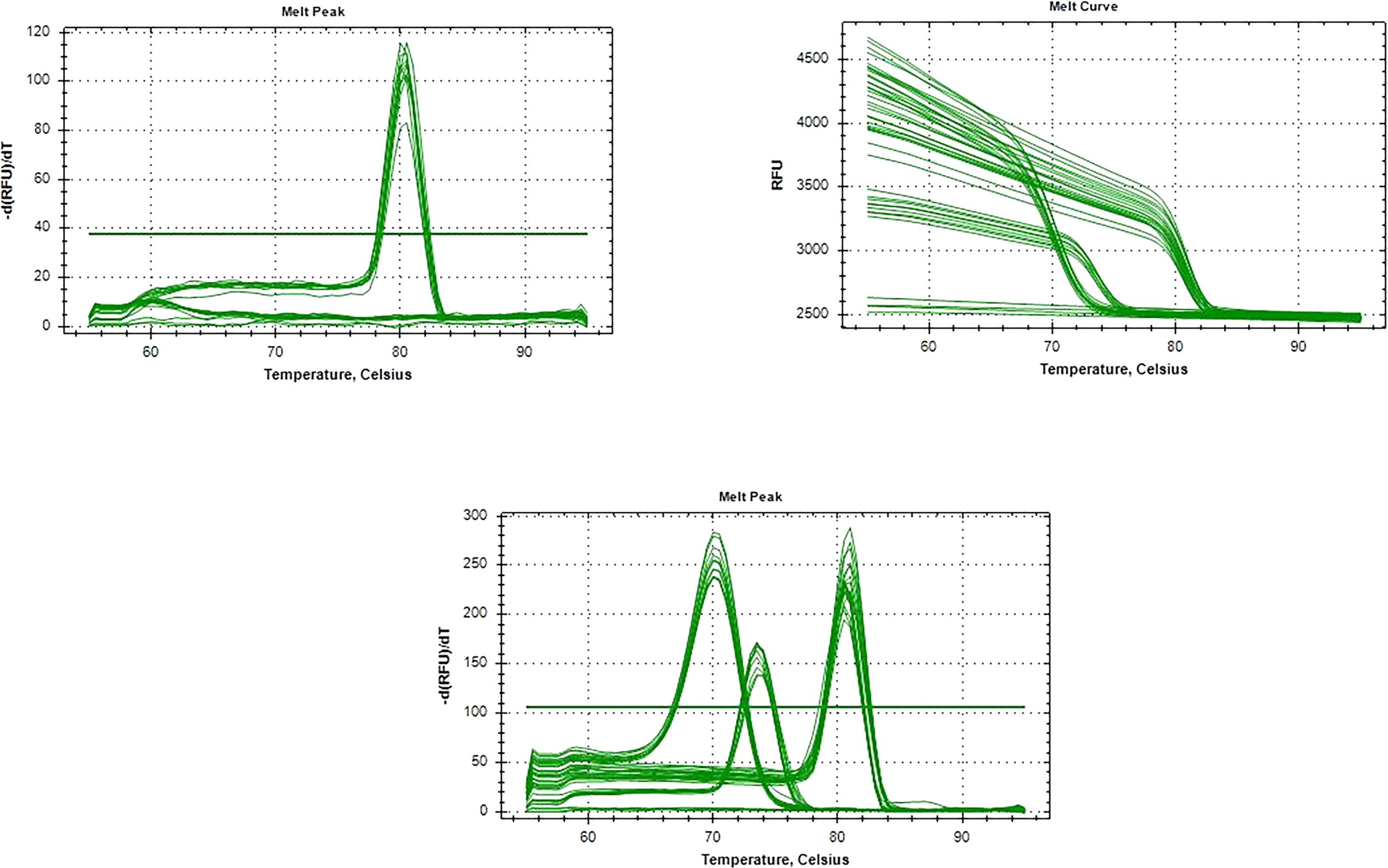

Supplement: Supplementary file 1 [file mmc1.docx]
