# Supplementary material for: Imidacloprid exposure cause the histopathological changes, activation of TNF-α, iNOS, 8-OHdG biomarkers, and alteration of caspase 3, iNOS, CYP1A, MT1 gene expression levels in common carp (Cyprinus carpio L.)
Source: Toxicol Rep. 2017 Dec 27;5:125–33. doi: 10.1016/j.toxrep.2017.12.019 (PMC5751999; doi:10.1016/j.toxrep.2017.12.019)
Supplement: Supplementary file 2 [file mmc2.docx]

| **Caspase 3** | **Groups** | | | | | |
| --- | --- | --- | --- | --- | --- | --- |
| Dosage | Cont | 24 h | 48 h | 72 h | 96 h | Levene sig. |
| Low dose | 0.60±0.003 | 0.7±0,01 | 0.81±0.005 | 0.98±0.06 | 1,1±0.05* | 0.06 |
| High dose | 0.60±0.003 | 0.82±0.05 | 0.88±0.05 | 1.1±0.04* | 1.29±0.005* | 0.06 |
| **iNOS** | **Groups** | | | | | |
| Dosage | Cont | 24 h | 48 h | 72 h | 96 h | Levene sig. |
| Low dose | 0.67±0.1 | 1.2±0.1* | 1.4±0.1* | 1.7±0.4** | 1.94±0.2** | 0.235 |
| High dose | 0.67±0.1 | 1.4±0.008* | 1.6±0.005* | 1.96±0.06** | 2.3±0.1*** | 0.235 |
| **CYP1A** | **Groups** | | | | | |
| Dosage | Cont | 24 h | 48 h | 72 h | 96 h | Levene sig. |
| Low dose | 0.44±0.02 | 0.65±0.01 | 0.74±0.02 | 0.87±0.01 | 1.1±0.05* | 0.171 |
| High dose | 0.44±0.02 | 0.77±0.01 | 0.87±0.005 | 0.95±0.02 | 1.2±0.01* | 0.171 |
| **MT1** | **Groups** | | | | | |
| Dosage | Cont | 24 h | 48 h | 72 h | 96 h | Levene sig. |
| Low dose | 0.64±0.05 | 0.82±0.01 | 0.92±0.01 | 0.96±0.008 | 1.2±0.005* | 0.813 |
| High dose | 0.64±0.05 | 0.93±0.008 | 1.2±0.08* | 1.2±0.08 * | 2.1±0.05** | 0.813 |

**Table S1: Showing changes of caspase 3, iNOS, CYP1A and MT1 gene expression levels in high and low dose IMI exposed to brain tissues.**

The results of caspase 3, iNOS, CYP1A and MT1 gene expressions are expressed as Mean ± SE. (**P<0.05*, ***P<0.01*, ****P<0.001*). Levene significance *P>0.05*.
